# Open questions on physical chemistry of crystal growth from congruent melts

**DOI:** 10.1038/s42004-021-00571-6

**Published:** 2021-09-16

**Authors:** Feier Hou

**Affiliations:** Department of Chemistry, Western Oregon University, Monmouth, OR USA

**Keywords:** Physical chemistry, Materials chemistry

## Abstract

Crystallization is observed in both nature and in the lab, and is critical to diverse areas of science and technology. Here, the author summarizes the theories that have been proposed to explain crystal growth from melts, and raises some open questions that remain.

Crystallization is critical to diverse areas of science and technology, ranging from geological phenomena, to chemical and materials synthesis, to energy and information storage technologies. There are many ways to grow crystals, such as from supersaturated solutions, from congruent melts (liquids of pure substances or mixtures that do not change their compositions when melting), and by chemical vapor deposition, to name a few. Among them, crystal growth from congruent melts has been observed since ancient times, with the most common example being water frozen into ice. Studies of crystal growth have largely focused on experimental methods^1^, but theories have also been developed to explain the physical chemistry of crystal growth. This Comment will focus on the open questions concerning the physical chemistry of crystal growth from congruent melts.

## Current theories

**Fig. 1:**
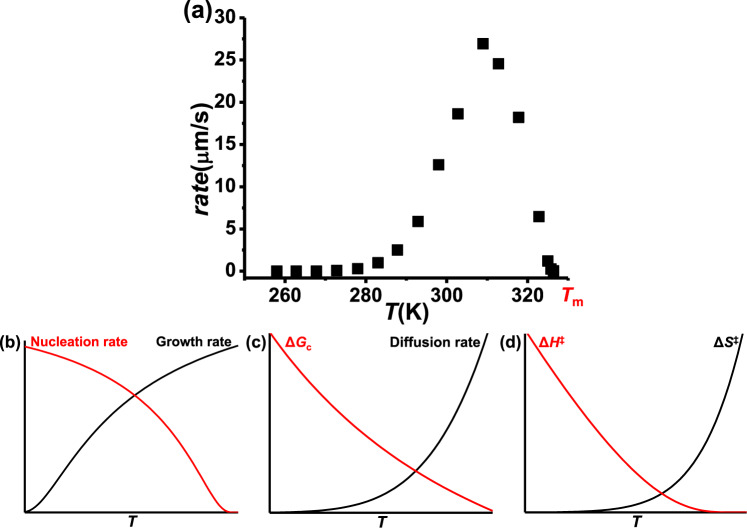
Experimental results and theoretical explanations of crystal growth from congruent melts. **a** The rate of crystal growth of o-terphenyl;^19^ (**b**), (**c**), and (**d**) are schematic representations of the parameters in CNT, ICG, and TZT that explain the rate of crystal growth, respectively. The curves in (**b**–**d**) are only schematic and not to scale.

It has been observed that the rate of crystallization from melts increases as temperature increases until it reaches a maximum at some temperature, *T*_max_, above which it decreases as temperature increases (“anti-Arrhenius”) until it reaches zero at the melting temperature, *T*_m_ (see Fig. 1a for an example). Several theories have been proposed to explain that behavior, but each has their limitations that will be discussed in the next section. One of them is the classical nucleation theory (CNT, Fig. 1b)^2–4^. Crystallization consists of two steps: nucleation, which is the formation of a partially ordered region of particles (nucleus) in a liquid; and growth, which is growing of the bulk crystal from the nucleus. According to CNT, as temperature increases, the rate of growth increases, while the rate of nucleation decreases. Below *T*_max_, nucleation is fast while growth is the rate-limiting step, thus the rate of crystallization increases as temperature increases; above *T*_max_, growth is fast, but nucleation is slow, which results in the decrease of the overall crystallization rate.

Another theory that has been proposed to explain crystallization is the interface-controlled growth model (ICG, Fig. 1c)^5–9^. According to ICG, the difference in the Gibbs free energy between the liquid and the crystal (Δ*G*_c_) is the driving force for crystallization, and it decreases as temperature increases and approaches *T*_m_; crystallization is also controlled by diffusion of liquid particles to attach to the interface between the liquid and the crystal, the rate of which increases as temperature increases. Below *T*_max_, Δ*G*_c_ is large but diffusion is slow; as temperature increases, diffusion rate increases, and the crystallization rate increases. Above *T*_max_, diffusion is fast, yet Δ*G*_c_ decreases as temperature approaches *T*_m_, which leads to the decrease in the crystallization rate.

Transition zone theory (TZT, Fig. 1d) has also been proposed to explain crystal growth from melts^10^. TZT extends transition state theory to condensed phase processes, where cooperative rearrangements of groups of particles are required^11^ instead of single molecules passing over an activation barrier, thus a transition zone exists as an analog to the transition state, which consists of groups of cooperative liquid particles with the configuration that leads to the formation of crystal. Furthermore, the enthalpy (Δ*H*^‡^) and entropy (Δ*S*^‡^) of activation of crystal growth are both temperature dependent, due to the temperature dependence of the entropy of the liquid and the size of the cooperative regions: at low temperatures, the liquid has fewer configurations accessible, thus its entropy is closer to the transition zone, and Δ*S*^‡^ is small negative; meanwhile, the cooperative regions in the liquid are large, thus more particles need to be transformed simultaneously in each activation event, which requires more energy, so Δ*H*^‡^ is large, which leads to a slow rate of crystal growth. As the temperature approaches *T*_m_, the cooperative regions in the liquid are smaller, thus Δ*H*^‡^ is lower, however, the liquid has many more configurations accessible, so Δ*S*^‡^ is larger negative, which also leads to a slow rate of crystal growth.

## Open questions and challenges

The physical chemistry of crystal growth from melts has been studied since the 1900s. So far, we know that on the atomic level, crystal growth involves many rearrangements of atoms in the melt (with only short-range order) to form the crystalline structure (with long-range order), which indicates the process is related to both the intermolecular forces and the entropy of the liquid. However, several open questions still remain.

### How do we improve the current theories?

Like all scientific theories, the theories proposed are our best explanations instead of the absolute truth. They each have limitations that need to be addressed; and with new experimental techniques, new evidence will surface, and they will likely need to be improved.

CNT treats nucleation and growth as parallel processes while singular nucleation events must occur before crystal growth, and contrary to CNT, anti-Arrhenius behavior has been observed in the rate of single crystal growth, without further nucleation, above *T*_max_^5,12^. Furthermore, it has been shown that for some substances, such as lithium disilicate^13^ and water^14^, CNT does not describe experimental results very well.

The ICG model assumes ΔG_c_ affects the rate of crystal growth, but ΔG_c_ is the difference in the Gibbs energy between the reactant (liquid) and the product (crystal), which is a thermodynamic property and should not affect kinetics of crystal growth; also, it assumes diffusion, which is proportional to the viscous relaxation of liquids, affects rate of crystallization, but relaxation of liquid is faster than the rate of crystallization and is generally complete before crystallization^15^. Furthermore, different types of interfaces (screw dislocation, 2-D, or normal) between crystal and melt have been proposed, but differentiating between different types of interfaces is largely based on which type fits the experimental data the best^16^, and there are no experimental observations for each type of interface, thus even though ICG fits experimental data reasonably well, the parameters are largely empirical and do not necessarily reflect the chemistry of the substances^17^.

Among the three theories, TZT provides the best fit to experimental data^17^. Furthermore, the Δ*H*^‡^ and Δ*S*^‡^ values from TZT are consistent with the physical and chemical properties of substances^10^. However, although TZT can explain the physical chemistry of crystal growth, it cannot predict the rate or Δ*H*^‡^ and Δ*S*^‡^ of crystal growth based on just the physical properties of the substances, besides, at least one of the parameters that describe the temperature dependence of Δ*S*^‡^ is still empirical^10^.

### What are the applications of the theories? Can we use them to make predictions?

The study of the physical chemistry of crystal growth does not stop at the theories. From a practical perspective, and as part of the scientific method, we need to use the theories to make predictions. What is the rate of crystal growth at a certain temperature for a given substance? For substances with polymorphism, which polymorph forms from the melt under a given condition? What are the possible crystal structures, and where are they on a liquid-solid phase diagram? What will be the structure of the crystals grown from a material synthesis? So far, those questions have been explored mostly experimentally, but recently, computational methods such as machine learning have been shown to be promising to help answer some of those questions^18^. Whether, and how, the above theories can be applied to predict the answers to those questions remains an open question.

### How generalizable is our knowledge on crystal growth from congruent melts to other types of crystal growth?

There are many other ways to grow crystals, during which different physical and chemical processes from those during crystal growth from melts may be involved. For example, to understand crystal growth from solutions, diffusion of solute particles should be considered. However, all crystal growth mechanisms are fundamentally the formation of a crystalline structure from reactants/media without long-range order, so there must be something in common between different types of crystal growth. How can our knowledge on crystal growth from congruent melts help us understand other types of crystal growth? Even though a “theory of everything” may be impossible, can we find better explanations to other types of crystal growth starting from the theories for crystal growth from melts?

### Challenges in answering the open questions

A major challenge in answering the above questions is that for crystal growth, it is difficult to completely remove the empiricism and find out what exactly happens on the atomic level. Although it is possible to visualize atoms with current techniques such as TEM, since crystal growth occurs in the condensed phase where atoms move cooperatively, changes in one atom can lead to changes of many other atoms, which all need to be tracked simultaneously. Also, atoms in the congruent melt flow, so the cooperative regions are constantly changing; meanwhile, at a given moment, the cooperative regions in a liquid are different due to the lack of long-range order in liquids. Therefore, a large sample of the melt needs to be observed, or the sample needs to be observed for a long time, and an ensemble or time average need to be taken to obtain the overall behavior of atoms during crystal growth. In addition, there are many different types of crystal growth, each of which has many different parameters that can affect crystal growth, so fundamental explanations to crystal growth requires complex, extensive studies of many different systems and types of crystal growth. All those tasks, although possible, are challenging and time-consuming, and may also require new experimental techniques to be developed.

Another challenge is the lack of emphasis on chemistry in condensed phases in undergraduate chemical education:^1^ most general chemistry classes focus on molecules and reactions in dilute media, and only briefly (if at all) mention solid state chemistry. Besides, at least in the USA, a solid state chemistry class is not always offered to undergraduate students, since it is not part of the ACS curriculum for Bachelor’s degree programs. As a result, students are more likely to think of chemistry as “molecular”, and their lack of familiarity with chemistry in condensed phases can lead to a shortage of workforce in the study of physical chemistry of crystal growth.

## Outlook

Several theories have been proposed to explain crystal growth from melts, yet open questions and challenges remain. The rapid development in advanced experimental techniques and computational methods will help answer the open questions. Meanwhile, we need to increase students’ familiarity with chemistry in the condensed phase to have a continuous workforce to study the physical chemistry of crystal growth.
